# Collaborative care programs for pregnant and postpartum individuals with opioid use disorder: Organizational characteristics of sites participating in the NIDA CTN0080 MOMs study

**DOI:** 10.1016/j.josat.2023.209030

**Published:** 2023-04-04

**Authors:** Frankie B. Kropp, Marcela C. Smid, Michelle R. Lofwall, Elisha M. Wachman, Peter R. Martin, Sean M. Murphy, Christine M. Wilder, T. John Winhusen

**Affiliations:** a Department of Psychiatry and Behavioral Neuroscience, University of Cincinnati College of Medicine, 3131 Harvey Avenue, Cincinnati, OH 45229, USA; b University of Utah, 50 N. Medical Drive, Salt Lake City, UT 84132, USA; c Departments of Behavioral Science and Psychiatry, University of Kentucky College of Medicine, 845 Angliana Avenue, Lexington, KY 40508, USA; d Boston Medical Center, 801 Albany Street, Boston, MA 02119, USA; e Vanderbilt Psychiatric Hospital, Vanderbilt University Medical Center, 1601 23rd Avenue South, Suite 3035, Nashville, TN 372124, USA; f Department of Population Health Sciences, Weill Cornell Medical College, 1300 York Avenue, New York, NY 10065, USA; g Center for Addiction Research, University of Cincinnati College of Medicine, 3230 Eden Avenue, Cincinnati, OH 45267, USA

**Keywords:** Opioid use disorder, Organizational factors, Collaborative care, Pregnant, Postpartum

## Abstract

**Introduction:**

Pregnant individuals with substance use disorders face complex issues that may serve as barriers to treatment entry and retention. Several professional organizations have established recommendations on comprehensive, collaborative approaches to treatment to meet the needs of this population, but information on real-world application is lacking. Sites participating in the NIDA CTN0080 “Medication treatment for Opioid use disorder in expectant Mothers (MOMs)”—a randomized clinical trial of extended release compared to sublingual buprenorphine among pregnant and postpartum individuals (PPI)—were selected, in part, because they have a collaborative approach to treating PPI with opioid use disorder (OUD). However, organizational differences among sites and how they implement expert recommendations for collaborative care could impact study outcomes.

**Methods:**

Prior to study launch at each of the 13 MOMs sites, investigators used the Pregnancy and Addiction Services Assessment (PAASA) to collect information about organizational factors. Input from a team of addiction, perinatal, and economic evaluation experts guided the development of the PAASA. Investigators programmed the PAASA into a web-based data system and summarized the resultant site data using descriptive statistics.

**Results:**

Study sites represented four US census regions. Most sites were specialty obstetrics & gynecology (OB/ GYN) programs providing OUD services (*n* = 9, 69.2 %), were affiliated with an academic institution (*n* = 11, 84.6 %), and prescribed buprenorphine in an ambulatory/outpatient setting (n = 11, 84.6 %); all sites offered access to naloxone. Sites reported that their population was primarily White, utilized public insurance, and faced numerous psychosocial barriers to treatment. Although all sites offered many services recommended by expert consensus groups, they varied in how they coordinated these services.

**Conclusions:**

By providing the organizational characteristics of sites participating in the MOMs study, this report assists in filling the current gap in knowledge regarding similar programs providing services to PPI with OUD. Collaborative care programs such as those participating in MOMs are uniquely positioned to participate in research to determine the most effective models of care and to determine how research can be integrated into those clinical care settings.

## 1. Introduction

The ongoing epidemic of opioid use in the United States over the past two decades has impacted many individuals. Pregnant individuals are no exception—the rate of deliveries complicated by opioid use disorder (OUD) has quadrupled with a concomitant rise in neonatal opioid withdrawal (NOWS) (Haight et al., 2018; Hayes & Brown, 2012; Jones et al., 2012; Patrick et al., 2017). Recommended best practice for the treatment of pregnant individuals with OUD utilizes medications for opioid use disorder (MOUD), including methadone and buprenorphine (BUP), combined with counseling and behavior therapies (American College of Obstetricians and Gynecologists, 2017; American Society of Addiction Medicine, 2020; Reddy et al., 2017; Substance Abuse and Mental Health Services Administration, 2018; World Health Organization, 2014b). While effective interventions for OUD exist and can improve outcomes for both the mother and infant, pregnant individuals with substance use disorders (SUDs) face significant challenges that may serve as a barrier to treatment entry and retention (Bedrick et al., 2020; Greenfield et al., 2007; Reising et al., 2019; Substance Abuse and Mental Health Services Administration, 2018; Wilder & Winhusen, 2015). Following delivery, individuals with OUD may face additional challenges that increase their vulnerability for return to non-prescribed use, overdose, and death in the first year postpartum (Schiff et al., 2018; Smid et al., 2020).

In response to the complex issues that pregnant and postpartum individuals (PPI) with OUD experience, the Substance Abuse and Mental Health Services Administration (SAMHSA), American College of Obstetricians and Gynecologists (ACOG), American Society of Addiction Medicine (ASAM), and World Health Organization (WHO) have established recommendations for a comprehensive, collaborative approach to treatment that brings together providers and services from multiple disciplines. Although the intricacies of each set of expert recommendations are beyond the scope of this article, in general the recommendations encompass: 1) Identifying and assessing pregnant individuals with OUD and other SUD by utilizing screening tools, psychosocial assessments, biological toxicology screens; 2) providing MOUD, including naloxone for opioid overdose, rather than opioid withdrawal; 3) providing specialized prenatal care, including physical examination, laboratory testing, imaging, increased pregnancy monitoring; 4) providing a full range of SUD treatment services, including individual and/or group counseling, case management, peer support specialists, mutual support groups, smoking cessation; 5) addressing comorbidities, including testing for infectious disease (HIV, Hepatitis B/C), screening and management of co-occurring medical and behavioral health conditions; 6) providing adequate peripartum pain management; 7) providing ongoing care postpartum, including ongoing treatment for OUD and other SUDs, contraceptive information and access, breast-feeding guidance; 8) providing assessment and care for infants with prenatal exposure to opioids, including management of NOWS, infant developmental assessment, well-baby care; and 9) addressing barriers to receiving services (American College of Obstetricians and Gynecologists, 2017; American Society of Addiction Medicine, 2020; Substance Abuse and Mental Health Services Administration, 2018; World Health Organization, 2014b). Models of care integrating both obstetric and behavioral health treatment increase engagement and improve perinatal outcomes in a cost-effective manner (Crane et al., 2019; Goler et al., 2008; Goodman et al., 2022; Johnson, 2019; Jones et al., 2008; Premkumar et al., 2019). However, we know little about the settings, services delivered, and staff/providers who are implementing this integration of multidisciplinary care for PPI with OUD and to what extent the expert recommendations are being followed. Previous studies suggest that understanding organizational factors such as population characteristics, staffing patterns, size and type of program, funding sources, geographic locations, and adherence to empirically established standards of care may assist in improving treatment delivery for persons with SUD (D’Aunno, 2006; Kimberly & McLellan, 2006; Kramlich et al., 2018; Krans et al., 2019; Meyer & Phillips, 2015; Short et al., 2018). Past research has identified differences in organizational factors as important to the translation of evidence-based treatments into clinical practice (Jacobson et al., 2020; McGovern et al., 2004; McLellan et al., 2003).

The NIDA Clinical Trials Network study “Medication treatment for Opioid use disorder in expectant Mothers (MOMs): a pragmatic randomized trial comparing extended-release and daily buprenorphine formulations (CTN0080)” (Winhusen et al., 2020) will evaluate the impact on mother and infant outcomes of treating OUD in PPI with extended-release (XR) BUP, compared to sublingual (SL) BUP. All MOMs sites use a collaborative care model for treating pregnant individuals with OUD, in which prenatal and MOUD services are either: 1) fully integrated into the program or 2) partially provided in-program with remaining services provided through close collaborations with other programs within the organization or provided via linkage/referral to an external provider. Sites participating in the study are diverse in terms of their organizational characteristics and how they implement expert recommendations, which could affect study outcomes. The aim of the current project is to characterize organizational factors and service-delivery methods of sites participating in a large multisite trial of MOUD for PPI.

## 2. Materials and methods

### 2.1. Development of the Pregnancy and Addiction Services Assessment (PAASA)

A review of the literature during protocol development failed to identify an instrument for characterizing models of care and other organizational factors for the management of PPI with OUD. Hence, the investigative team developed the Pregnancy and Addiction Services Assessment (PAASA) to characterize the organizational factors associated with the MOMs sites. Investigators reviewed several surveys used to characterize organizational and treatment factors in health care settings associated with SUDs, most notably the Better Outcomes Through Research for Newborns (BORN) survey (Bogen et al., 2017) and the Addiction Treatment Inventory (ATI) (Carise et al., 2000). Members of the study executive team who were experts in research and treatment of PPI with SUD created a comprehensive set of questions specific to the treatment of PPI with OUD, which were piloted with substance use treatment providers, revised, and sent to experts in addictions research and treatment, maternal/fetal medicine, and economic analysis serving on the CTN0080 MOMs protocol development team for review and revision. Developers added specific funding and staffing items to assist with the study’s planned economic analysis. The final version of the survey was programmed into REDCap (Patridge & Bardyn, 2018), hosted by the Center for Clinical and Translational Science and Training at the University of Cincinnati. The survey contained up to 55 questions, depending upon skip patterns, including questions about organizational characteristics, population served, staff characteristics, MOUD type offered, and service delivery. The PAASA addressed most, but not all, expert recommendations for comprehensive care of PPI with OUD. Investigators did not address expert recommendations around peripartum pain management and management of NOWS in the PAASA as these were more relevant to delivery and neonatal care units than to the outpatient programs participating in the study. Most questions were single-answer, although others asked the respondent to check all that applied or to provide total number or percentages. Questions around the provision of MOUD addressed integrated services within the site only. Six questions asked the respondent to identify the OB/GYN, psychosocial addiction treatment, psychiatric/mental health, general medical, and other services provided to patients, and then to indicate which method each used: 1) integrated within the program, 2) collaborative with providers elsewhere within the same organization, or 3) available only through linkage or referral to an outside source. The full survey is available in Appendix 1.

### 2.2. Data collection

Investigators chose participating sites for the MOMs study based on the extent to which they: 1) provide BUP to pregnant individuals in an office-based setting, 2) offer BUP treatment following delivery for ≥12 months, and 3) have sufficient clinic enrollment to support the target study randomization rate. To be considered a good candidate for the study, a site must have indicated utilizing a model of care in which close collaboration existed between prenatal care and addiction treatment providers and, where possible, treatment was integrated. Twenty-two potential sites submitted Site Selection Surveys, resulting in the selection of 12 sites to participate in the study; the most common reasons for site exclusion were: 1) inadequate enrollment of BUP-SL-maintained pregnant individuals; 2) minimal clinical trials experience; and/or 3) minimal resources to implement the trial (additional information regarding site selection available in Winhusen et al., 2020). The study added a thirteenth site approximately 18 months later. Prior to study initiation, investigators emailed a link to the online survey to representatives from each of the initial 12 sites participating in the CTN0080 MOMs study; the thirteenth site was provided with the link just prior to opening the site for enrollment. Sites will complete the PAASA a second time near the end of participant data collection; the current report describes results from the initial survey only. Respondents reported only on the array of services in their program that are provided to PPI with OUD. Investigators defined “the program” as those services that are included in the organized specialty clinic or billing unit that provides services to PPI with OUD, regardless of whether the physical locations of these services are the same. Upon completion, study staff reviewed surveys for missing data and asked sites to complete missing data points.

### 2.3. Data analysis

Investigators employed descriptive statistics to summarize PAASA survey responses for all sites, and grouped services according to the expert recommendation with which they were associated. As some sites reported multiple methods for service delivery, investigators summarized responses to those questions based on the most integrative method used by the site (In-Program = Most integrative; Linkage/Referral = Least integrative). The PAASA questions restricted types of MOUD provided to in-program services only.

### 2.4. Human subjects protection

The main protocol for the CTN0080 MOMs study included administration of the PAASA. The UC College of Medicine Institutional Review Board reviewed and approved this study in accordance with principles outlined in the Declaration of Helsinki.

## 3. Results

The original 12 sites selected for the MOMS study completed the PAASA in July and August 2019; the study added a thirteenth site, which completed the PAASA in May 2022. All 13 MOMs sites completed the PAASA in its entirety.

### 3.1. Geographic characteristics

Based on the US Census regional divisions (U.S. Census Bureau, 2022), 5 of the 13 participating sites (38.5 %) were in the West census region (2 Mountain Division, 3 Pacific Division). Four sites (30.8 %) represented the South census region (3 South Atlantic Division, 1 East South-Central Division), and 3 sites (23.1 %) represented the Northeast census region (2 New England Division, 1 Middle Atlantic Division). The remaining site (7.7 %) was in the Midwest census region (East North Central Division). Most programs (*n* = 8, 61.5 %) were in a major metropolitan area (population 1 million or more), with 3 (23.1 %) located in a large city (population 300,000–999,000) and 2 (15.4 %) were located in a moderately large city (population 100,000–299,999).

### 3.2. Organizational characteristics

The sites varied greatly in the number of pregnant individuals with OUD served per year, with a median (IQR) of 50 (35–155). Most sites (*n* = 9, 69.2 %) were specialty OB/GYN programs for persons with SUDs, with the remainder being specialty addiction programs (*n* = 3, 23.1 %) or primary care programs for PPI with SUD (*n* = 1, 7.7 %). Seven of the sites (53.8 %) were hospital ambulatory clinics associated with a multi-site healthcare organization. Of the remaining sites, 3 (23.1 %) were freestanding medical hospitals, 2 (15.4 %) were part of a multi-site SUD treatment organization, and 1 (7.7 %) was a freestanding ambulatory program. Eleven of the 13 sites (84.6 %) were affiliated with an academic health center. On average, sites indicated that 89.3 % (S.D. 11.1) of their PPI patients with OUD were insured by public insurance (Medicare, Medicaid). Sites reported a median (IQR) waiting period of 2 (0–4) days for intake for new pregnant individuals with OUD.

### 3.3. PPI characteristics

Table 1 provides information on the percentage of racial and ethnic identity reported within the PPI served by the sites. Table 2 provides information on barriers to care found among sites’ PPI.

### 3.4. Provider characteristics

The 13 sites involved in the CTN0080 study employed a wide variety of clinicians and staff, including physicians (OB/GYN, Maternal Fetal Medicine [MFM], psychiatrists, addiction medicine specialists, other), other medical clinicians (nurse practitioners, physician assistants, other nurses), nonmedical clinicians (psychologists, social workers, addictions and mental health counselors), and peer support specialists to provide comprehensive medical, psychosocial, and recovery care services. On average, physicians contributed 1.7 (S.D. 1.2) full-time equivalents (FTEs) to the sites, while other medical clinicians contributed an average of 2.4 (S.D. 1.4) FTEs. Nonmedical clinicians contributed an average of 1.8 (S.D. 1.3) FTEs. On average, sites utilized 5.3 (S.D. 2.9) physicians to prescribe BUP, while 6 sites (46.2 %) also employed nurse practitioners and 2 sites (15.4 %) employed physician assistants to prescribe BUP.

### 3.5. Implementation of expert recommendations

**Fig. 1.**
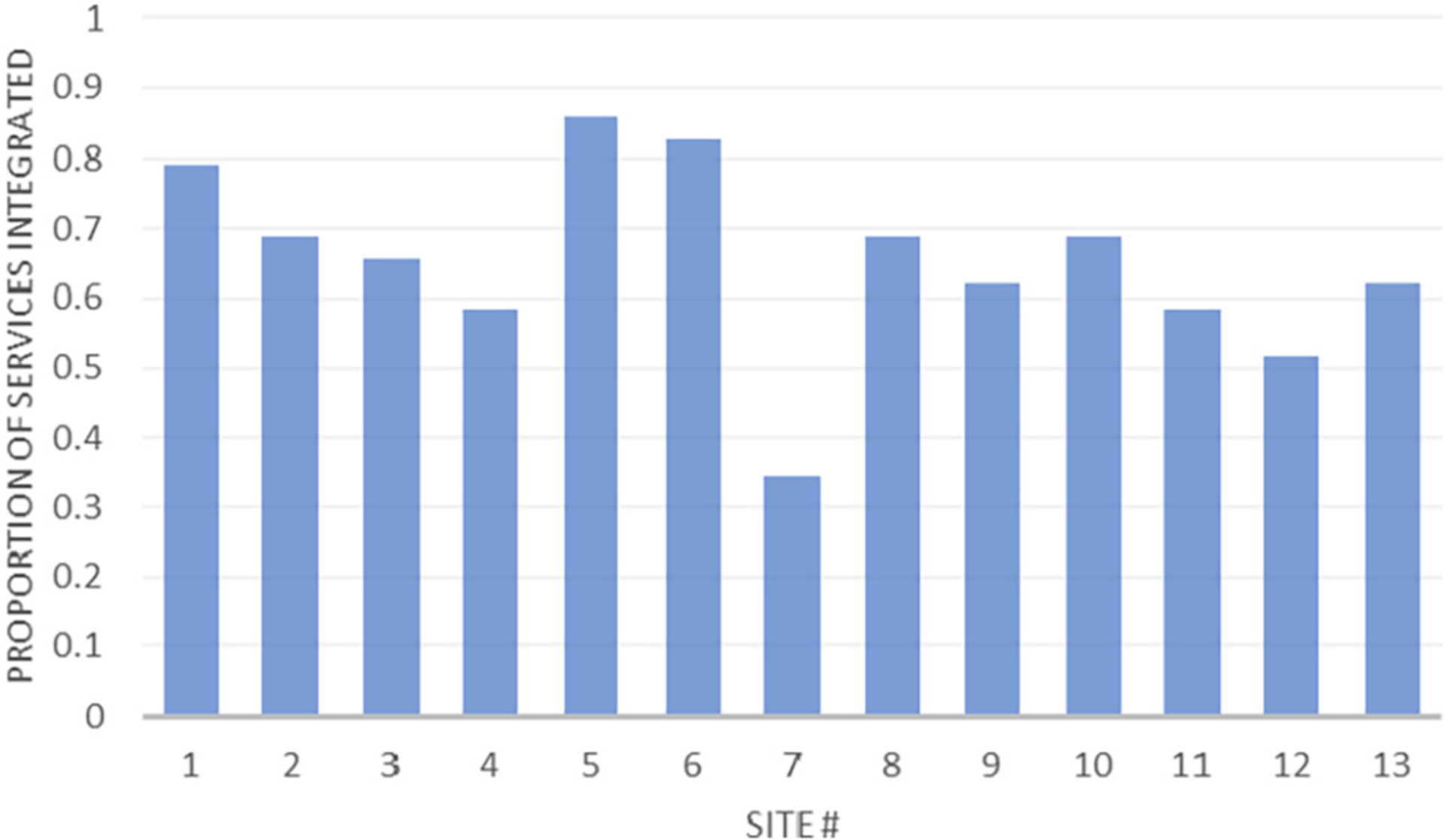
Proportion of recommended services integrated into care as a function of site.

Study sites integrated many components of the expert recommendations into their programs; as needed, sites coordinated with other clinics within their organization or with external organizations to ensure comprehensive care for their patients. Analysis of sites indicated some degree of variability in how services were integrated into patient care; sites integrated from 10 to 25 of the 29 recommended clinical services assessed by the PAASA into their programs. Table 3 provides detailed information on each site’s method of implementing recommended clinical services; the table includes only services which were specifically mentioned in one or more of the 4 guidance documents. MOUD items reflect integrated MOUD services only. Fig. 1 shows the proportion of recommended non-MOUD clinical services that are fully integrated into care as a function of site. Table 4 provides information about how each site provided additional services to address barriers to treatment.

#### 3.5.1. Identify PPI with OUD and other SUD

All sites reported screening for illicit substance use utilizing both psychosocial assessments and urine drug screens. Some sites provided screening for driving under the influence (n = 3, 23.1 %) and utilized breathalyzers for alcohol screening (n = 5, 38.5 %) as well.

#### 3.5.2. Provide MOUD

Four (30.8 %) of the 13 sites dispensed methadone through their licensed methadone treatment program (OTP) in addition to providing BUP; 4 (30.8 %) sites also provided naltrexone to patients who were already being treated with naltrexone at the time they became pregnant. Eleven sites (84.6 %) provided both BUP-only (BUP mono) products and products combining BUP and naloxone (BUP/NX), while 1 site (7.7 %) provided only the BUP mono product and 1 site (7.7 %) provided only the BUP/NX product. Three sites (23.1 %) provided injectable BUP and 2 sites (15.4 %) provided implantable BUP. Two sites (15.4 %) dispensed BUP through their OTP while the remaining 11 (84.6 %) sites prescribed BUP in a non-OTP ambulatory/outpatient clinic setting. Most sites performed BUP induction by administering one or more initial doses in person (inpatient or in-clinic), with additional induction doses prescribed/dispensed for home administration. All sites provided patients with naloxone for overdose treatment, with 11 (84.6 %) providing access at the time of program admission; 6 (46.2 %) dispensed naloxone and 7 (53.8 %) provided prescriptions only. Six sites (46.2 %) also dispensed naloxone or provided prescriptions to family members and/or significant others.

#### 3.5.3. Provide specialized prenatal care

At minimum, all sites provided physical examinations to their pregnant individuals; only 2 sites (15.4 %) did not include ongoing prenatal care as part of their program. In addition to the services shown in Table 3, 8 sites (61.5 %) provided in-program birthing classes and 6 sites (46.2 %) reported integrated fetal genetic testing and diagnostic services.

#### 3.5.4. Provide comprehensive substance use disorder treatment

Most sites (*n* = 12, 92.3 %) offered services in an ambulatory/ outpatient setting. Additionally, 4 (30.8 %) offered intensive outpatient (IOP) or partial hospitalization (PH), and 6 (46.2 %) offered inpatient or residential services. Group services reported by sites included psycho-educational groups, relapse-prevention groups, and addiction-focused group counseling and/or psychotherapy. About half of the sites (*n* = 7, 53.8 %) reported providing in-program peer support. Three sites (23.1 %) offered family education and/or counseling in addition to services listed in Table 3.

#### 3.5.5. Address comorbid disorders

Four sites (30.8 %) provided primary care services to address co-occurring health concerns, and all but one site (*n* = 12, 92.3 %) provided testing for infectious diseases commonly associated with substance use such as HIV and hepatitis B/C. Most sites (*n* = 10, 76.9 %) provided in-program assessment for mental/behavioral health disorders, while the remaining 3 sites coordinated this service elsewhere within their organization. Considerable variation existed in how sites coordinated mental health services for their patients, although most (*n* = 10, 76.9 %) indicated management of psychiatric medications within their programs.

#### 3.5.6. Provide postpartum care

Most sites (*n* = 10, 76.9 %) offered all recommended postpartum services as part of their programs. These included postpartum visits, instruction and counseling around breastfeeding, and family planning/ contraceptive services. In addition to services indicated in Table 3, 11 (84.6 %) sites integrated postpartum gynecological care into their programs.

#### 3.5.7. Assess and care for opioid-exposed infants

The PAASA did not include questions specifically aimed at specialized services for opioid-exposed infants or assessment of neonatal opioid withdrawal. However, PAASA questions asked sites to indicate their provision of pediatric services. Sites varied in their approach to services for infants, with 5 sites (38.5 %) providing in-program “well-baby” care and 3 sites (23.1 %) offering developmental assessment services within their program.

#### 3.5.8. Address barriers to services

Sites varied considerably in how they assisted patients with resources to reduce barriers to care. About half of the sites (*n* = 7, 53.8 %) provided in-program assistance with transportation to clinic appointments; assistance in applying for government resources such as Medicaid, Medicare, or Social Security; and with basic needs such as food, clothing, or infant care supplies. Fewer sites (*n* = 5, 38.5 %) assisted with providing childcare during clinic visits, while 4 sites assisted patients without insurance in obtaining services through waiving of fees or covering costs through contributions from donors. Six sites (46.2 %) provided assistance with housing needs: 1 site provided emergency shelter only; 2 sites provided long-term recovery/supportive housing only; and 3 sites provided both emergency and long-term housing.

## 4. Discussion

This description of organizational factors associated with sites participating in the CTN0080 MOMs study assists in filling the current gap in knowledge regarding academic health and community SUD programs providing services to PPI with OUD. It provides a glimpse into differences among the way that these services are coordinated. More precisely, this analysis demonstrates the wealth of information that these comprehensive and experienced programs offer to furthering research on integrative/collaborative care models and methods for implementing expert recommendations for treating this population. For example, a post-hoc analysis is tentatively planned in the MOMs study to explore site characteristics associated with treatment outcome.

Given that previous research has identified significant challenges facing PPI with OUD in accessing care, we find it encouraging that results from the PAASA indicate that study sites, which were selected in part because of the comprehensive nature of their services, offer many elements described in the coordinated collaborative care models proposed by expert consensus groups (American College of Obstetricians and Gynecologists, 2017; American Society of Addiction Medicine, 2020; Substance Abuse and Mental Health Services Administration, 2018; World Health Organization, 2014b). Even these exemplary sites, however, were unable to integrate the full range of recommended services within their programs. Further, the number of integrated services did not appear to be associated with the type of program. The site integrating the largest number of clinical services (Site #5) was one of the nonacademic specialty SUD treatment programs. The site integrating the largest number of services to address barriers (Site #7) was a specialty OB/GYN program. Given that these exemplary sites do not fully integrate all recommended services, a collaborative care model may be as effective as an integrative care model. If true, the use of effective collaborations could assist programs in maximizing resources to ensure that the PPIs they serve effectively meet all expert recommendations regardless of whether they are able to integrate them into their in-program service delivery. A 2017 executive summary of a workshop by the National Institute of Child Health and Human Development identified numerous research gaps and opportunities for screening and management of PPI with OUD (Reddy et al., 2017). Research instruments collecting information about specific services and how they are coordinated, such as the PAASA, can assist in exploring which array of services are most critical for PPI with OUD, and how best to implement those models. Programs implementing collaborative care models, such as the ones in this study, are uniquely positioned to provide recruitment sites for further research on these questions.

The finding that most sites had very low rates of racial and ethnic minority patient populations is consistent with recent findings of disparity in receipt of MOUD, particularly buprenorphine, during pregnancy for persons of color compared with White, non-Hispanic individuals (Hansen et al., 2013; Krans et al., 2019; Lagisetty et al., 2019; Schiff et al., 2020). Such disparities limit the generalizability of results to the full population of PPI with OUD and indicate barriers that must be addressed for sites participating in randomized clinical trials. In the CTN0080 MOMs study, sites are specifically focusing on recruitment and retention efforts that assist in breaking down barriers for persons of color.

Opioid overdose is a leading cause of death in PPI (Bagley et al., 2018; Mehta et al., 2016; Schiff et al., 2018; Smid et al., 2019; Smid et al., 2020); and overdose education with naloxone distribution is recommended for the prevention of opioid overdose (American Society of Addiction Medicine, 2016; US Department of Health and Human Services, 2018; World Health Organization, 2014a). Although all sites provide access to naloxone for all PPI with OUD in their program, some do not offer it as part of the standard intake process, and many of those that do offer access at intake do so using prescriptions that must be filled elsewhere. To reduce the risk of opioid overdose in PPIs with OUD, further research should determine effective methods for increasing direct access to naloxone as they present for care.

The findings presented here have limitations. Most notably, the 13 sites participating in CTN0080 MOMs were chosen in large part due to their strong collaborative care model, significant treatment resources, and experience with clinical trials; we do not know to what extent they represent other programs in the United States that are treating the population of PPI with OUD. Further, the sample size is small (*n* = 13) and 11 of the 13 sites were specialized practices affiliated with academic health centers serving a large number of PPIs. Consequently, the majority of sites in this study represent a narrowly focused practice type rather than representing the broad range of program structures serving this population. Because of these limitations, readers should take caution in interpreting the findings. In addition, the information collected provides relatively sparse details on the organizations involved. Prior research in examining organizational factors associated with substance use treatment has typically utilized a mixed-methods approach, combining extensive surveys with interviews and other qualitative methods. By relying solely on this brief survey, the current data provide only a high-level view of the participating sites.

## 5. Conclusion

The current article provides some preliminary information about types of providers and staff along with their respective effort/time allocations, services offered, and population served within academic health centers and large community-based SUD treatment organizations that are providing treatment for PPI with OUD using a collaborative care model, which is lacking in the existing literature. Sites selected to participate in the CTN0080 MOMs study are providing a comprehensive array of medical, counseling, harm reduction (e.g., naloxone), case management, and psychosocial recovery support services in accordance with recommendations for multidisciplinary comprehensive care and are taking steps to reduce barriers to care for this population. Further research should more fully explore the organizational factors utilized in care for PPI with OUD to determine the most effective/cost-effective models of care and how best to implement and expand access to those models. Collaborative care programs, such as those participating in the CTN0080 MOMs study, are uniquely positioned to serve as recruiting sites for such research.

## Supplementary Material

1

## Figures and Tables

**Table 1:** PPI race/ethnicity characteristics – all sites.

Number of sites reporting % of population served (*n* = 13)	0 %	1 %−10 %	11 %−25 %	26 %−50 %	50 %−75 %	76 % or greater
African American/Black	0	9	3	1	0	0
American Indian/Alaskan Native	6	7	0	0	0	0
Asian	7	6	0	0	0	0
Native Hawaiian/other Pacific Islander	9	4	0	0	0	0
White/Caucasian	0	0	1	0	4	8
More than one race/bi-racial	0	12	0	1	0	0
Hispanic	2	10	0	0	0	1

**Table 2:** Barriers to care found among sites’ PPI.

Barrier type Number and % of sites reporting	All sites (n = 13)	
	n	%
Homelessness	12	92.3 %
Chronic severe mental illness	13	100.0 %
History of trauma	13	100.0 %
Non-English speaking	2	15.3 %
On probation or parole	13	100.0 %
Pending legal charges	13	100.0 %
Unable to pay for services	10	76.9 %
Active domestic violence	13	100.0 %
Transportation problems	13	100.0 %
Major medical illness	11	84.6 %
Lack of childcare	11	84.6 %

**Table 3:** Site-level adherence to expert recommendations for clinical services.

Recommendation	Site												
	1	2	3	4	5	6	7	8	9	10	11	12	13
Assess for OUD and Other SUD													
Psychosocial assessment	I	I	I	I	I	I	I	I	I	I	I	I	I
DUI assessment	R	R	R	N	I	I	N	N	R	R	I	C	R
Urine drug screening	I	I	I	I	I	I	I	I	I	I	I	I	I
Breathalyzer	I	R	R	N	I	I	N	N	R	C	I	I	R
Provide MOUD (in-program only)													
Methadone	I	N	N	N	I	I	N	I	N	N	N	N	N
Buprenorphine	I	I	I	I	I	I	I	I	I	I	I	I	I
Naltrexone with caution	I	N	I	N	N	N	N	I	I	N	N	N	N
Naloxone distribution	I	I	I	I	I	I	I	I	I	I	I	I	I
Provide specialized prenatal care													
Ongoing prenatal care	I	I	I	I	I	I	C	I	I	I	R	I	I
Physical exam	I	I	I	I	I	I	I	I	I	I	I	I	I
Increased monitoring for high risk	I	I	I	I	I	I	C	I	I	I	R	I	I
Lab testing	I	I	I	I	I	I	C	I	I	I	C	I	I
Ultrasound	I	C	I	I	R	I	C	C	C	I	R	I	I
Provide comprehensive psychosocial substance use disorder treatment													
Individual counseling	I	I	I	R	I	I	C	I	C	I	I	I	I
Group counseling	I	I	I	R	I	I	C	I	C	C	I	I	I
Case management	I	I	I	I	I	I	I	I	I	I	I	I	I
Peer support	R	N	I	N	I	I	I	C	I	R	I	I	R
Mutual support groups	R	R	C	R	I	I	R	R	R	R	I	R	R
Smoking cessation	I	I	I	C	I	I	I	I	I	I	I	I	I
Address co-occurring conditions/disorders													
Infectious disease testing	I	I	I	I	I	I	I	I	I	I	I	C	I
Primary care services	I	I	C	C	R	I	C	C	N	C	R	C	I
Mental health assessment	I	I	C	I	I	C	I	I	I	I	I	C	I
Individual MH therapy	I	I	C	I	I	I	C	I	C	I	I	C	R
Group MH therapy	N	C	I	N	I	C	C	R	C	C	I	C	C
MH case management	R	C	I	I	I	I	C	I	I	I	R	C	R
Psychiatric crisis services	C	C	C	C	I	C	C	I	I	I	R	C	R
Psychiatric medication management	I	I	I	I	N	C	I	I	I	I	I	C	I
Integrated SUD-MH	I	C	C	I	I	C	C	I	C	I	I	C	C
Provide postpartum care													
Postpartum visits	I	I	I	I	I	I	C	I	I	I	R	I	I
Breastfeeding guidance	I	I	I	I	I	I	I	I	I	I	R	C	I
Family planning/contraception	I	I	I	I	I	I	C	I	I	I	C	I	I
Assess and care for opioid-exposed infants													
Well-baby care	I	I	C	C	I	I	C	C	I	C	R	C	C
Infant developmental assessment	I	I	C	C	R	I	C	C	C	C	R	C	R

I = integrated into program; C = collaboration with provider elsewhere within organization; R = linkage/referral to external resource; N = not reported.

**Table 4:** Site-level services to overcome barriers.

Service	Site												
	1	2	3	4	5	6	7	8	9	10	11	12	13
Transportation assistance	R	C	I	I	I	I	I	C	I	R	C	R	I
Financial assistance	C	C	R	R	R	I	I	C	R	C	I	R	I
Childcare	R	C	I	N	I	C	I	R	I	R	I	R	R
Access to government resources	C	C	I	I	R	I	I	I	C	C	C	I	I
Basic needs	R	C	I	R	I	I	I	I	I	C	C	C	I
Housing	R	R	I	R	I	I	I	I	C	R	I	C	R

I = integrated into program; C = collaboration with provider elsewhere within organization; R = linkage/referral to external resource; N = not reported.
