# Supplementary material for: Collaborative care programs for pregnant and postpartum individuals with opioid use disorder: Organizational characteristics of sites participating in the NIDA CTN0080 MOMs study
Source: J Subst Use Addict Treat. Author manuscript; Available in PMC 2023 Jun 8. (PMC10249488; doi:10.1016/j.josat.2023.209030)
Supplement: 1 [file NIHMS1904508-supplement-1.pdf]

PREGNANCY AND ADDICTION SERVICES ASSESSMENT (PAASA)

Assessment of Services for Pregnant and Post-Partum Women with Opioid Use Disorder

You have been identified as the contact person at your program who is best able to provide information about your organization and the services you deliver. Information we receive from you will be combined with information from other service providers; no information identifying your specific program will be presented in a report, publication, or presentation.

The questions in this survey pertain only to the array of services in your program that are provided to pregnant and post-partum women with opioid-use disorder. If your program provides services to other populations, such as pregnant women *without* opioid use disorder, please do not include those populations when responding to the survey questions. By “program”, we mean those services that are included in the organized specialty clinic or program billing unit which provides services to pregnant/post-partum women with opioid use disorder. Even if the physical location of some aspect of the service is different, it will be considered part of your program as long as it is included under the same clinic or billing unit. Other services received by your patients that are under a different clinic or billing unit within your organization should be considered as “outside” your program, even if they are located elsewhere within your organization.

Please navigate through the survey using the buttons at the bottom of each page; please do not use the "back" button/symbol on your web browser. We ask that you answer every question. We know your time is valuable - we believe the survey will take only about 15 - 20 minutes for you to complete. If you feel you are not the best person to provide this information, please forward the email with the link to the person you believe should complete the survey. If possible, we would appreciate receiving your responses within 7 business days.

Thank you so much for all you do to help ensure that women receive the services they need for good outcomes for themselves and their children! We appreciate you taking the time to share your information with us.

A. ORGANIZATIONAL CHARACTERISTICS

The following questions will assist us in understanding your organization. Please select all responses that apply.

A-1. In which state is your program located (select one)?

- ☐ Alabama
- ☐ Alaska
- ☐ Arizona
- ☐ Arkansas
- ☐ California
- ☐ Colorado
- ☐ Connecticut
- ☐ Delaware
- ☐ District of Columbia
- ☐ Florida
- ☐ Georgia
- ☐ Hawaii

- ☐ Idaho
- ☐ Illinois
- ☐ Indiana
- ☐ Iowa
- ☐ Kansas
- ☐ Kentucky
- ☐ Louisiana
- ☐ Maine
- ☐ Maryland
- ☐ Massachusetts
- ☐ Michigan
- ☐ Minnesota
- ☐ Mississippi
- ☐ Missouri
- ☐ Montana
- ☐ Nebraska
- ☐ Nevada
- ☐ New Hampshire
- ☐ New Jersey
- ☐ New Mexico
- ☐ New York
- ☐ North Carolina
- ☐ North Dakota
- ☐ Ohio
- ☐ Oklahoma
- ☐ Oregon
- ☐ Pennsylvania
- ☐ Puerto Rico
- ☐ Rhode Island
- ☐ South Carolina
- ☐ South Dakota
- ☐ Tennessee
- ☐ Texas
- ☐ Utah
- ☐ Vermont
- ☐ Virginia
- ☐ Washington
- ☐ West Virginia
- ☐ Wisconsin
- ☐ Wyoming

A-2. In what type of community setting is your clinic/program located (select one)?

- ☐ Major metropolitan area (pop. 1 million or more)
- ☐ Large city (pop. 300,000 to 999,999)
- ☐ Moderately large city (pop. 100,000 to 299,999)
- ☐ Large town (pop. 20,000 to 99,999)
- ☐ Small town (pop. 200 to 19,999)
- ☐ Village or other small incorporation (pop. less than 200)
- ☐ Rural area – unincorporated
- ☐ Tribal reservation
- ☐ Other; specify: ____

A-3. How would you characterize the primary focus of your program (select all that apply)?

- ☐ Primarily an OB/GYN or Maternal/Fetal Health program
- ☐ Primarily an Addiction Treatment program
- ☐ Primarily a Primary Care (Family Medicine, Internal Medicine, Pediatrics) program
- ☐ Specialty OB/GYN or Maternal/Fetal Health program for women with substance use disorders
- ☐ Specialty Addiction Treatment program for pregnant/post-partum women
- ☐ Specialty Primary Care program for pregnant/post-partum women with substance use disorders
- ☐ Other; specify: ____

A-4. To what extent does your program provide integrated OB and Addiction services (select one)?

- ☐ Both OB and Addictions services are provided within the program
- ☐ OB services are provided within the program and have a greater focus; some Addictions services are provided within the program but to a lesser degree
- ☐ OB services are provided within the program; Addictions services are provided by a partnering clinic or progam within our organization
- ☐ OB services are provided within the program; Addictions services are provided through linkage/referral to community addiction providers outside our organization
- ☐ Addiction services are provided within the program and have a greater focus; some OB services are provided within the program but to a lesser degree
- ☐ Addiction services are provided within the program; OB services are provided by a partnering clinic or program within our organization
- ☐ Addiction services are provided within the program; OB services are provided through linkage/referral to community OB providers outside our organization

A-5. In what type of organization does your program operate (select one)?

- ☐ Freestanding ambulatory program
- ☐ Freestanding medical hospital or other healthcare inpatient facility
- ☐ Freestanding inpatient/residential addiction program
- ☐ Freestanding inpatient/residential mental health program
- ☐ Multi-site healthcare organization
- ☐ Multi-site addictions treatment organization
- ☐ Multi-site mental health treatment organization
- ☐ Veterans Administration (VA) Healthcare
- ☐ Criminal justice/corrections facility
- ☐ Indian Health Service
- ☐ Tribal health organization (not IHS)
- ☐ Community public health organization
- ☐ Other; specify: ____

A-6. Is your program affiliated with an academic institution (college/university)?

- ☐ YES
- ☐ NO

A-7. What accreditation is held by your program (select all that apply)?

- ☐ The Joint Commission (TJC)
- ☐ Commission on Accreditation of Rehabilitation Facilities (CARF)
- ☐ Clinic/program does not hold any accreditation
- ☐ Other accreditation; specify: ____

A-8. Approximately what proportion of your pregnant/postpartum patients have the following insurance type?

Private insurance: _____________

Medicaid/Other public insurance: _____________

Self pay/Uninsured: _________________

Other; specify: ____

A-9. About how many pregnant women with opioid use disorder does your program treat each year? If your program has more than one site, please respond for this site only. ______________

A-10. About how long is the average wait period for a new patient intake for pregnant women with opioid use disorder (in days)? ________

A-11. On average, how many months do your specialty services for pregnant and/or postpartum women continue after delivery? ________

A-12. On average, how long is the OB post-partum follow up period at your program?________

B. PATIENT POPULATION SERVED

Please describe the *usual* patient population served by your program.

B-1. What types of pregnant/postpartum patients are served by your program (select all that apply)?

- ☐ Pregnant adult females with Opioid Use Disorder only
- ☐ Pregnant adult females with Opioid Use Disorder PLUS other alcohol/drug use disorder(s)
- ☐ Pregnant adolescent (ages 17 and younger) females with Opioid Use Disorder only
- ☐ Pregnant adolescent (ages 17 and younger) females with Opioid Use Disorder PLUS other alcohol/drug disorder(s)
- ☐ Post-partum adult females with Opioid Use Disorder only
- ☐ Post-partum adult females with Opioid Use Disorder PLUS other alcohol/drug use disorder(s)
- ☐ Post-partum adolescent (ages 17 and younger) females with Opioid Use DIsorder only
- ☐ Post-partum adolescent (ages 17 and younger) females with Opioid Use DIsorder PLUS other alcohol/drug use disorder(s)

B-2. What other characteristics are found among your usual patient population (select all that apply)?

- ☐ Homelessness

- ☐ Chronic severe mental illness
- ☐ History of trauma
- ☐ Does not speak English
- ☐ On probation/parole
- ☐ Adjudicated to treatment/medical care
- ☐ Pending legal charges
- ☐ Unable to pay for services, no insurance coverage
- ☐ Veteran
- ☐ Non-US citizens/foreign nationals
- ☐ Active domestic violence
- ☐ Transporation problems
- ☐ Major medical illness
- ☐ Lack of childcare

B-4. Approximately what percentage of your patient population self-identifies as Hispanic? ___________

B-5. Approximately what percentage of your patient population self-identifies as:

	**0%**	**1% - 10%**	**11% - 25%**	**26% - 50%**	**50% - 75%**	**76% or greater**
African-American/Black?	☐	☐	☐	☐	☐	☐
American Indian/Alaskan Native?	☐	☐	☐	☐	☐	☐
Asian?	☐	☐	☐	☐	☐	☐
Native Hawaiian/Other Pacific Islander?	☐	☐	☐	☐	☐	☐
White/Caucasian?	☐	☐	☐	☐	☐	☐
More than one race/Bi-racial?	☐	☐	☐	☐	☐	☐

C. STAFF CHARACTERISTICS

Please indicate *unduplicated* provider/staff information for this program only. In case of multiple credentials for a provider/staff member, please indicate only the highest credential or the credential most representative of his/her work in this location.

C-1. Medical staff. Please indicate the total number and the % effort dedicated to the program. For example, someone who works full-time in the program should be listed at 100% effort.

	**Total number**	**% Effort**
General Obstetricians/ Gynecologists (MD/DO)		
Maternal Fetal medicine, OB specialists (MD/DO)		
Addictionologists (MD/DO - Board certified in Addiction Medicine or Addiction Psychiatry)		
Psychiatrists (MD/DO - not board certified in Addictions)		
Neonatologists (MD/DO)		
Other Pediatricians (MD/DO)		
Other physicians (MD/DO - Internal medicine, family practice, etc)		
Physician Assistants		
Nurse Practitioners/Advance Practice RNs		
Registered Nurses (Bachelor's degree or above)		
Registered Nurses (less than a Bachelor's degree)		
Other licensed nurses		
Midwives		
Doulas		

C-2. Non-medical staff. Please indicate the total number and the % effort dedicated to the program. For example, someone who works full-time in the program should be listed at 100% effort.

	**Total number**	**% Effort**
Psychologists (PhD)		
Independently Licensed Mental Health providers (e.g., Social Workers, Counselors, etc)		
Independently Licensed Addiction Counselors		
Mental Health providers, not independently licensed		
Addiction Counselors, not independently licensed		
Peer Counselors/Facilitators		
Other non-medical provider staff (please specify type, #, % effort)		

C-3. How many of the staff in your program hold DEA waivers for buprenorphine?

	# Providers
Physicians	______________________
Physician Assistants	______________________
Nurse Practitioners	______________________

D. MEDICATIONS USED

Methadone

D-1. Do you administer/dispense Methadone in the treatment of pregnant women with Opioid Use Disorder?

- ☐ YES (Continue)
- ☐ NO (Skip to D-8)

Methadone Utilization

D-2. In what setting does initial dose escalation for pregnant patients take place (Select one)?

- ☐ Inpatient setting.
- ☐ Outpatient setting.
- ☐ Both inpatient and outpatient settings are used

  D-2a: If both inpatient and outpatient settings are used, what determines which setting is used? (specify): ____

D-3. How is initial dose escalation for pregnant patients managed (Select one)?

- ☐ Standard escalation protocol used with all patients, regardless of pregnancy status.
- ☐ Standard escalation protocol developed for pregnant patients only.
- ☐ No standard escalation protocol; escalation determined by individual patient characteristics.

D-4. Please indicate the way in which Methadone is used for pregnant women with Opioid Use Disorder in your program (Select one):

- ☐ Methadone Maintenance treatment (licensed Opioid Treatment Program) ONLY
- ☐ Detoxification (licensed Opioid Treatment Program) ONLY (Skip to D-8)
- ☐ Both Maintenance and Detoxification (licensed Opioid Treatment Program)
- ☐ Hospital administration (not licensed Opioid Treatment Program) to maintain or detoxify opioid-dependent patients for medical purposes as allowed by law (21CFR 1306.07) ONLY (Skip to D-8)
- ☐ Other; specify: ____

D-5. Does your program utilize "Split-Dosing" for pregnant patients?

- ☐ Yes, for all pregnant patients
- ☐ Yes, but only as individual patient needs dictate
- ☐ No

D-6. How often does your program perform methadone peak and trough labs with pregnant patients (select one)?

- ☐ Once - 1st Trimester
- ☐ Once - 2nd Trimester
- ☐ Once - 3rd Trimester
- ☐ 2 times during pregnancy
- ☐ More than 2 times during pregnancy
- ☐ Do not regularly perform Peak and Trough labs based on pregnancy alone
- ☐ Do not perform Peak and Trough labs at all

D-7. What is the *minimum* recommended requirement for pregnant patients on methadone to attend psychosocial addiction service delivery (e.g., individual/group counseling, education groups, etc.) in your program (Select one)?

- ☐ More than 3 times per week
- ☐ 2 - 3 times per week
- ☐ Once per week
- ☐ 2-3 times per month
- ☐ Once per month
- ☐ Less than once per month
- ☐ Depends solely on patient’s phase/length of time in treatment; no modification for pregnant patients
- ☐ No minimum recommended requirement for psychosocial services/individualized schedule according to patient needs and preferences
- ☐ Other (specify):____

Buprenorphine

D-8. Do you utilize Buprenorphine in the treatment of pregnant women with Opioid Use Disorder?

- ☐ YES (Continue)
- ☐ NO (Skip to D-21)

Buprenorphine Prescribers

D-9. Please indicate which practitioners prescribe buprenorphine to pregnant women (select all that apply):

- ☐ General Obstetricians/Gynecologists (MD/DO)
- ☐ Maternal Fetal medicine specialists (MD/DO)
- ☐ Addictionologists (MD/DO - board certified in Addiction Medicine or Addiction Psychiatry)
- ☐ Psychiatrists (MD/DO - not board certified in Addiction Psychiatry)
- ☐ Neonatologists (MD/DO)
- ☐ Other Pediatricians (MD/DO)
- ☐ Other Physicians (MD/DO - Internal medicine, family practice, etc, not board certified in Addiction Medicine)
- ☐ Physician Assistants
- ☐ Nurse Practitioners/Advance Practice RNs

Buprenorphine Utilization

D-10. Please indicate the ways in which Buprenorphine is utilized with pregnant women (select all that apply):

D-10a. <u>Buprenorphine/naloxone combined product</u>:

- ☐ Buprenorphine/naloxone is dispensed as part of a licensed Opioid Treatment Program (42CFR Part 8)
- ☐ Buprenorphine - sublingual/buccal is prescribed for in-clinic administration ONLY
- ☐ Buprenorphine - sublingual/buccal is prescribed for home administration ONLY
- ☐ Buprenorphine/naloxone is prescribed for both in-clinic and home administration

D-10b. <u>Buprenorphine-only products</u>:

- ☐ Buprenorphine is dispensed as part of a licensed Opioid Treatment Program (42CFR Part 8)
- ☐ Buprenorphine - sublingual/buccal is prescribed for in-clinic administration ONLY
- ☐ Buprenorphine - sublingual/buccal is prescribed for home administration ONLY
- ☐ Buprenorphine is prescribed for both in-clinic and home administration
- ☐ Probuphine implants are surgically administered in-clinic
- ☐ Probuphine implants are prescribed for surgical administration in a different medical facility
- ☐ Injectable long-acting buprenorphine is administered in-clinic

D-11. How are inductions onto sublingual/buccal Buprenorphine (+/-naloxone) managed in your program (select all that apply)?

- ☐ Inductions are performed entirely in an inpatient setting.
- ☐ Some induction dosing is administered in an inpatient setting, while additional doses are prescribed/dispensed for home administration post-discharge.
- ☐ Induction dosing is administered on an outpatient basis, with all doses administered in-clinic.
- ☐ Some induction dosing is administered in-clinic on an outpatient basis, while additional doses are prescribed/dispensed for home administration.
- ☐ All induction dosing is administered at home.
- ☐ The induction procedure changes based on estimated gestational age at time of induction. Specify:____
- ☐ Other; please specify: ____

D-12. In general, how many visits are planned for purposes of induction for each pregnant patient?

- ☐ 1 - to obtain the medication prescription only; home induction protocol is then used.
- ☐ 1 - initial dosing occurs in-clinic; once stabilized, induction is completed at home.
- ☐ 2.
- ☐ 3 or more.

D-13. In general, how long (in minutes) is a visit planned for purposes of induction for each pregnant patient?

____

D-13a. Does this change postpartum? If so, how?

____

D-14. Please indicate the number of each type of provider that generally participates in a visit planned for purposes of induction for each pregnant patient?

	**Total #**
General Obstetricians/ Gynecologists (MD/DO)	
Maternal Fetal medicine, OB specialists (MD/DO)	
Addictionologists (MD/DO - Board certified in Addiction Medicine or Addiction Psychiatry)	
Psychiatrists (MD/DO - not board certified in Addictions)	
Other physicians (MD/DO - Internal medicine, family practice, etc)	
Physician Assistants	
Nurse Practitioners/Advance Practice RNs	
Registered Nurses (Bachelor's degree or above)	
Registered Nurses (less than a Bachelor's degree)	
Other licensed nurses	

	**Total #**
Psychologists (PhD)	
Independently Licensed Mental Health providers (e.g., Social Workers, Counselors, etc)	
Independently Licensed Addiction Counselors	
Mental Health providers, not independently licensed	
Addiction Counselors, not independently licensed	
Peer Counselors/Facilitators	

Other non-medical provider staff (please specify type and #)

D-14a. Does this change postpartum? If so, how?

____

D-15. In general, how long (in minutes) is a medication management visit for a pregnant patient on buprenorphine (+/-naloxone)?

____

D-15a. Does this change postpartum? If so, how?

____

D-16. Please indicate the number of each type of provider that generally participates in a medication management visit for a pregnant patient on sublingual/buccal buprenorphine?

	**Total #**
General Obstetricians/ Gynecologists (MD/DO)	
Maternal Fetal medicine, OB specialists (MD/DO)	
Addictionologists (MD/DO - Board certified in Addiction Medicine or Addiction Psychiatry)	
Psychiatrists (MD/DO - not board certified in Addictions)	
Other physicians (MD/DO - Internal medicine, family practice, etc)	
Physician Assistants	
Nurse Practitioners/Advance Practice RNs	
Registered Nurses (Bachelor's degree or above)	
Registered Nurses (less than a Bachelor's degree)	
Other licensed nurses	

	**Total #**
Psychologists (PhD)	
Independently Licensed Mental Health providers (e.g., Social Workers, Counselors, etc)	
Independently Licensed Addiction Counselors	
Mental Health providers, not independently licensed	
Addiction Counselors, not independently licensed	
Peer Counselors/Facilitators	

Other non-medical provider staff (please specify type and #)

D-16a. Does this change postpartum? If so, how?

____

D-17. If Probuphine or injectable buprenorphine is administered in-clinic, in general, how long (in minutes) is a visit for a pregnant patient?

____

D-17a. Does this change postpartum? If so, how?

____

D-18 Please indicate the number of each type of provider that generally participates in a Probuphine or injectable buprenorphine administration visit for a pregnant patient?

	**Total #**
General Obstetricians/ Gynecologists (MD/DO)	
Maternal Fetal medicine, OB specialists (MD/DO)	
Addictionologists (MD/DO - Board certified in Addiction Medicine or Addiction Psychiatry)	
Psychiatrists (MD/DO - not board certified in Addictions)	
Other physicians (MD/DO - Internal medicine, family practice, etc)	
Physician Assistants	
Nurse Practitioners/Advance Practice RNs	
Registered Nurses (Bachelor's degree or above)	
Registered Nurses (less than a Bachelor's degree)	
Other licensed nurses	

	**Total #**
Psychologists (PhD)	
Independently Licensed Mental Health providers (e.g., Social Workers, Counselors, etc)	
Independently Licensed Addiction Counselors	
Mental Health providers, not independently licensed	
Addiction Counselors, not independently licensed	
Peer Counselors/Facilitators	

Other non-medical provider staff (please specify type and #)

D-18a. Does this change postpartum? If so, how?

____

D-19. How often does your program perform buprenorphine peak and trough labs with pregnant patients (select one)?

- ☐ Once - 1st Trimester
- ☐ Once - 2nd Trimester
- ☐ Once - 3rd Trimester
- ☐ 2 times during pregnancy
- ☐ More than 2 times during pregnancy
- ☐ Do not regularly perform peak and trough labs based on pregnancy alone
- ☐ Do not perform peak and trough labs at all

D-20. What is the *minimum* recommended requirement for pregnant patients on buprenorphine to attend psychosocial addiction service delivery (e.g., individual/group counseling, education groups, etc.) in your clinic/program (Select one)?

- ☐ More than 3 times per week
- ☐ 2 - 3 times per week
- ☐ Once per week
- ☐ Every 2 weeks
- ☐ Once per month
- ☐ Less than once per month
- ☐ Depends solely on patient's phase/length of time in treatment; no modification for pregnant patients
- ☐ No requirement for pregnant patients to attend psychosocial addictions services

Naltrexone

D-21. Do you utilize Naltrexone in the treatment of pregnant women with Opioid Use Disorder?

- ☐ YES (Continue)
- ☐ NO (Skip to D-25)

Naltrexone Utilization

D-22. Please indicate the ways in which Naltrexone is utilized with pregnant women (select all that apply)

- ☐ Oral naltrexone is prescribed for home administration
- ☐ Injectable long-acting naltrexone (e.g., Vivitrol) is administered in-clinic

D-23. Under what conditions do you utilize Naltrexone in pregnant women (select all that apply)?

- ☐ Naltrexone is used if the patient was already receiving Naltrexone at the time when pregnancy was discovered.
- ☐ Naltrexone is used with pregnant women who are re-entering treatment following incarceration or other long-term abstinence in a controlled environment.
- ☐ Naltrexone is used when there is some significant barrier to utilizing an opioid agonist/partial agonist treatment medication (such as severe allergy or legal mandate).
- ☐ Naltrexone is used when patients express a preference for this treatment option.

D-24. What is the *minimum* recommended requirement for pregnant patients on naltrexone to attend psychosocial addiction service delivery (e.g., individual/group counseling, education groups, etc.) in your program (Select one)?

- ☐ More than 3 times per week
- ☐ 2 - 3 times per week
- ☐ Once per week
- ☐ 2-3 Times per month
- ☐ Once per month
- ☐ Less than once per month
- ☐ Depends solely on patient's phase/length of time in treatment; no modification for pregnant patients
- ☐ No requirement for pregnant patients to attend psychosocial addictions services

Naloxone

D-25. Does your clinic prescribe or dispense naloxone for overdose treatment?

- ☐ YES (Continue)
- ☐ NO (Skip to E-1)

Naloxone Utilization

D-26. Please indicate the ways in which naloxone overdose prevention kits are provided to pregnant patients (select all that apply):

- ☐ Kit supplied directly to patient upon admission
- ☐ Kit supplied directly to patient upon request
- ☐ Kit supplied directly to patient following an overdose episode
- ☐ Prescription supplied directly to patient upon admission
- ☐ Prescription supplied directly to patient upon request
- ☐ Prescription supplied directly to patient following an overdose episode

☐ Kit supplied directly to patient's family/significant others

☐ Prescription supplied directly to patient's family/significant others

☐ Referral to a nearby resource for naloxone

☐ Other; please specify: ____________________

D-27. What type of training in the use of naloxone kits is provided by your program (select all that apply)?

☐ Verbal explanation by clinic/program staff

☐ Demonstration by clinic/program staff

☐ Provision of informational brochures/handouts

☐ Review of training video

☐ No training in use of naloxone kits provided to patient

☐ Other; please specify: ____________________

E. SERVICE DELIVERY

Please complete the questions below to assist us in understanding the services provided for patients in your program.

E-1. Please indicate your typical hours of operation.

		Not Open	Open 24 hours	Early morning (4 am - 8am)	Morning (8am - Noon)	Noon	Afternoon (Noon - 5pm)	Early Evening (5pm - 7pm)	Evening (7pm - 10pm)	Night (10pm - 4am)
MONDAY	FROM	☐	☐	☐	☐	☐	☐	☐	☐	☐
	TO	☐	☐	☐	☐	☐	☐	☐	☐	☐
TUESDAY	FROM	☐	☐	☐	☐	☐	☐	☐	☐	☐
	TO	☐	☐	☐	☐	☐	☐	☐	☐	☐
WEDNESDAY	FROM	☐	☐	☐	☐	☐	☐	☐	☐	☐
	TO	☐	☐	☐	☐	☐	☐	☐	☐	☐
THURSDAY	FROM	☐	☐	☐	☐	☐	☐	☐	☐	☐
	TO	☐	☐	☐	☐	☐	☐	☐	☐	☐
FRIDAY	FROM	☐	☐	☐	☐	☐	☐	☐	☐	☐
	TO	☐	☐	☐	☐	☐	☐	☐	☐	☐
	FROM	☐	☐	☐	☐	☐	☐	☐	☐	☐

SATURDAY	TO	☐	☐	☐	☐	☐	☐	☐	☐	☐
SUNDAY	FROM	☐	☐	☐	☐	☐	☐	☐	☐	☐
	TO	☐	☐	☐	☐	☐	☐	☐	☐	☐
Other (please specify):										

For this next set of questions, please indicate the services provided for pregnant/post-partum women with OUD, and whether those services are provided within your program (In Program), coordinated with other programs/providers within your organization (Coordinated Within Organization), or provided through linkage/referral to outside programs/providers (Linkage/Referral).

E-2. Please indicate the OB/GYN services provided for patients in your program (select all that apply):

**Service**	**In Program**	**Coordinated Within Organization**	**Linkage/ Referral**
Prenatal care visits	☐	☐	☐
Routine GYN services (pelvic exams, PAP, sexual health, etc.)	☐	☐	☐
Family planning/contraception	☐	☐	☐
High-risk pregnancy care	☐	☐	☐
Fetal diagnosis and genetic counseling	☐	☐	☐
Pregnancy lab testing	☐	☐	☐
Pregnancy ultrasound/sonograms and fetal monitoring	☐	☐	☐
Birthing classes/groups	☐	☐	☐
Breastfeeding instruction/coaching	☐	☐	☐
Labor and delivery (MD)	☐	☐	☐
Labor and delivery (midwife/doula)	☐	☐	☐
Post-partum visits	☐	☐	☐
Other; please specify ________________________________	☐	☐	☐

E-3. Please indicate the types of Addiction service formats provided for patients in your program (select all that apply):

Service Formats	In Program	Coordinated Within Organization	Linkage/ Referral
Ambulatory/Outpatient	☐	☐	☐
Intensive Outpatient (3 or more days per week, 3 hours or more per day)	☐	☐	☐
Partial Hospitalization (5 or more days per week, 6 hours or more per day)	☐	☐	☐
Inpatient (7 days per week, 24 hours per day, up to 28 days)	☐	☐	☐
Residential (7 days per week, 24 hours per day, 29 days or greater)	☐	☐	☐
Other; please specify ____	☐	☐	☐

E-4. Please indicate the Addiction (non-medication) services provided for patients in your program (select all that apply):

Service	In Program	Coordinated Within Organization	Linkage/ Referral
Psychosocial Assessment	☐	☐	☐
DUI Assessment	☐	☐	☐
Urine drug screens	☐	☐	☐
Breathalyzer/Other alcohol testing	☐	☐	☐
Psychoeducation groups	☐	☐	☐
12-Step/Self-Help groups	☐	☐	☐
Relapse Prevention groups	☐	☐	☐
Other addiction group counseling	☐	☐	☐
Group Psychotherapy (addiction focused)	☐	☐	☐
Individual Addiction Counseling	☐	☐	☐
Motivational Enhancement Therapy	☐	☐	☐
Cognitive Behavioral Therapy for addictions	☐	☐	☐
Acceptance and Commitment Therapy for addictions	☐	☐	☐
Trauma-focused Therapy for addictions	☐	☐	☐

Individual Psychotherapy (addiction focused)	☐	☐	☐
Marital/Couples Addiction Counseling	☐	☐	☐
Family Psychoeducation (addiction focused)	☐	☐	☐
Family Therapy (addiction focused)	☐	☐	☐
Family Groups (addiction focused)	☐	☐	☐
Smoking Cessation Treatment	☐	☐	☐
Case Management	☐	☐	☐
Inpatient Detoxification	☐	☐	☐
Outpatient Detoxification	☐	☐	☐
Peer support/recovery coaching	☐	☐	☐
Other; please specify ______________________________	☐	☐	☐

E-5. Please indicate the Psychiatric/Mental Health services provided for patients in your program (select all that apply):

**Service**	**In Program**	**Coordinated Within Organization**	**Linkage/ Referral**
Psychiatric/Mental Health Assessment	☐	☐	☐
Individual Psychotherapy/Counseling (mental health focused)	☐	☐	☐
Group Psychotherapy/Counseling (mental health focused)	☐	☐	☐
Mental Health Case Management	☐	☐	☐
Psychiatric Crisis Management Services	☐	☐	☐
Psychiatric Medication Management	☐	☐	☐
Domestic Violence/Trauma Psychotherapy/Counseling	☐	☐	☐
Co-occuring disorders (Addictions + other Mental Health) Integrated Services	☐	☐	☐
Other; please specify ______________________________	☐	☐	☐

E-6. Please indicate the other Medical services provided for patients in your program (select all that apply).

Service	In Program	Coordinated Within Organization	Linkage/ Referral
General medical labs (non-pregnancy)	☐	☐	☐
EKG	☐	☐	☐
Infectious disease testing (HIV, HBV, HCV, etc)	☐	☐	☐
Routine medical history and physical	☐	☐	☐
Primary care	☐	☐	☐
Routine vaccinations	☐	☐	☐
Well-baby care	☐	☐	☐
Routine pediatric care (non-infant)	☐	☐	☐
Pediatric developmental assessment	☐	☐	☐
Other; please specify ________________________________	☐	☐	☐

E-7. Please indicate any other services provided for patients in your program (select all that apply):

Service	In Program	Coordinated Within Organization	Linkage/ Referral
Parenting Education Classes	☐	☐	☐
Parenting Support/Counseling Groups	☐	☐	☐
Transportation Assistance for program services	☐	☐	☐
Childcare for program services	☐	☐	☐
Financial Assistance (service delivery free of charge, sponsored contributions, etc)	☐	☐	☐
Assistance with applying for state/federal resources (Medicaid/Medicare, SSI, etc)	☐	☐	☐
Basic Needs Assistance (food, clothing, baby supplies, etc)	☐	☐	☐
Emergency Housing/Temporary Shelter	☐	☐	☐
Supportive/Recovery Housing - Adults only	☐	☐	☐

Supportive/Recovery Housing - Parents and children	☐	☐	☐
GED preparation	☐	☐	☐
Needle/syringe exchange	☐	☐	☐
Drug take-backs	☐	☐	☐
Other; please specify ______________________________	☐	☐	☐

F. OTHER INFORMATION

F-1. Please feel free to tell us anything else you believe would help us in understanding your program and the services you provide.

Comments:

Thank you for participating this survey!
